# Fattening of Polish Holstein-Friesian × Limousin Bulls under Two Production Systems and Its Effect on the Fatty Acid Profiles of Different Fat Depots

**DOI:** 10.3390/ani11113078

**Published:** 2021-10-28

**Authors:** Monika Sobczuk-Szul, Magdalena Mochol, Zenon Nogalski, Paulina Pogorzelska-Przybyłek, Martyna Momot

**Affiliations:** Department of Cattle Breeding and Milk Evaluation, Faculty of Animal Bioengineering, University of Warmia and Mazury in Olsztyn, Oczapowskiego 5/137, 10-958 Olsztyn-Kortowo, Poland; khb@uwm.edu.pl (M.M.); zena@uwm.edu.pl (Z.N.); paulina.pogorzelska@uwm.edu.pl (P.P.-P.); martyna.momot@uwm.edu.pl (M.M.)

**Keywords:** beef, fatty acids, fat depot, production system

## Abstract

**Simple Summary:**

Cattle raised under different production systems differ in their ability to accumulate adipose tissue. Whereas the deposition of intramuscular fat is usually considered beneficial, excessive amounts of subcutaneous and internal fat are undesirable. This study compared the fatty acid (FA) profiles of four types of fat depots from crossbred bulls (Polish Holstein-Friesian × Limousin, PHF × LM) fattened semi-intensively (SI) and intensively (I). Intramuscular fat was most abundant in total polyunsaturated fatty acids (PUFAs), n-3 and n-6 PUFAs, and functional fatty acids C 18:2, C 18:3, C 20:4 and C 20:5 in comparison with the remaining fat types. Furthermore, note that external fat was more abundant in conjugated linoleic acid (CLA) than other fat types. This finding may have implications for both the beef industry and, due to the observed differences in FA composition, human health.

**Abstract:**

This study was designed to compare the fatty acid (FA) profiles of four types of fat depots from crossbred bulls (Polish Holstein-Friesian × Limousin, PHF × LM) fattened semi-intensively (SI) and intensively (I). Intramuscular fat was most abundant in total polyunsaturated fatty acids (PUFAs), n-3 and n-6 PUFAs, and functional fatty acids C 18:2, C 18:3, C 20:4 and C 20:5 in comparison with the remaining fat types. Furthermore, note that external fat was more abundant in conjugated linoleic acid (CLA) than other fat types. Grass silage fed to cattle during fattening had a beneficial influence on the FA profile of beef, and an increase in the amount of concentrate in the ration did not decrease beef quality.

## 1. Introduction

Recent years have witnessed increasing interest in the quality of beef rather than factors such as the growth rate of cattle or carcass composition, which used to be the main focus of producers. Grass-fed beef has been gaining popularity because it supports trends towards more extensive land use and delivers health benefits due to its favorable fatty acid (FA) profile, including elevated proportions of conjugated linoleic acid (CLA) and n-3 FAs [1,2].

Fat can exert both positive and negative effects on human health, depending on its FA composition [3]. Fat deposited in various regions of the carcass can have different FA profiles [4,5,6,7,8]. Intramuscular fat content of cattle is influenced by factors such as breed, sex, age and housing system but also the individual genetic background of an animal [9,10,11]. Lately, it has been shown that the ELOVL6 gene regulates the lipid metabolism and encodes a crucial protein that participates in lipogenesis by catalyzing the elongation of monounsaturated and saturated fatty acids [12]. The FA composition of adipose tissue is also determined by the nutritional regime of cattle and the length of the fattening period [13,14].

Fattening methods differ from farm to farm, and fattening intensity is determined by on-farm feeding capability, production profitability and market demand. Traditional and semi-intensive (SI) cattle production systems are most popular in Poland. In comparison with intensive (I) production systems, meat produced under SI systems has low levels of saturated FAs (SFAs) and high concentrations of n-3 FAs and CLA [15,16].

The aim of this study was to evaluate the effect of two cattle production systems on the FA profiles of different fat depots. The analyzed production systems are widespread in Poland. Meat from Polish Holstein-Friesian (PHF) cattle and their crosses predominates on the domestic market.

## 2. Materials and Methods

The study was conducted upon the approval of the Ethics Committee of the University of Warmia and Mazury (decision No. 121/2010). The experimental materials comprised 16 crossbred bulls produced by crossing PHF cows with Limousin (LM) bulls. Control fattening began at 7 months of age, after a 1-month adaptation period, and it lasted until 18 months of age.

The animals were divided into two groups, and they were fattened semi-intensively (SI) and intensively (I). The Total Mixed Ration (TMR), composed of grass silage and concentrate (triticale, rapeseed meal (RSM), premix), was available ad libitum. Two types of concentrate with different protein content were used (Table 1). The concentrates were formulated based on the amounts of protein digested in the small intestine, PDIN and PDIE [17]. Depending on the production system, the ratio of concentrate to grass silage (on a dry matter basis) was 25:75 (SI) and 40:60 (I) (Table 2). The ratios were adjusted every 4 weeks based on regular silage analysis. Bulls with BW below 300 kg received TMR containing concentrate I (25% RSM), and bulls with BW above 300 kg received TMR containing concentrate II (19% RSM).

The animals were slaughtered at the completion of fattening. All slaughter and post-slaughter processes were carried out in accordance with the current meat industry regulations (Council Regulation (EC) No. 1099/2009 of 24.09.2009 on the protection of animals at the time of killing). Half-carcasses were weighed, and conformation and fatness were evaluated based on the EUROP system criteria by a trained grader [18]. Ninety-six hours postmortem, three-rib (10th–12th rib) sections were sampled from the right half-carcasses (two cuts through a half-carcass, perpendicular to the spine, between the 9th and 10th, and the 12nd and 13rd thoracic vertebrae). Samples of four fat types were also collected: intramuscular, intermuscular, external and internal. Intramuscular fat samples were collected from *m. longissimus dorsi*, between the 10th and 12nd thoracic vertebrae. Intermuscular fat samples were collected from the round of beef, between the silverside and topside muscles. External fat samples were taken from the top of striploin, and internal fat samples were taken from the kidney fat depot region. Three-rib cuts were dissected, and the percentage content of soft tissues (lean meat, fat and tendons) and bones was determined. Fat samples were ground, and fat was extracted by the Soxhlet method with hexane as a solvent (Büchi B-811 extraction system, Flawil, Switzerland). Total crude fat content and the proportions of individual FAswere determined in accordance with the relevant standards [19,20]. Fatty acid methyl esters were obtained by dissolving the extracted fat in a mixture of methanol, chloroform and sulfuric acid, followed by methylation according to the Peisker method [21]. The percentage share of the analyzed FAs was determined by gas chromatography using the Varian CP 3 800 system (Varian, Palo Alto, CA, USA) with a split/splitless injector and a flame-ionization detector (FID). Samples (1 µL) of FA methyl esters were placed on a CP-Sil 88 capillary column (length: 100 m, inner diameter: 0.25 mm). All analyses were performed under identical conditions. The results were processed using the GALAXIE Chromatography Data System and FAs were identified by comparing their retention times with those of commercially available reference standards (Supelco, Inc., Sigma Aldrich, Bellefonte, PA, USA). Fatty acids were divided into the following groups: saturated fatty acids (SFAs) and unsaturated fatty acids (UFAs), including mono- and polyunsaturated fatty acids (MUFAs and PUFAs). The following ratios were calculated: UFA/SFA, MUFA/SFA, PUFA/SFA and n-6/n-3 PUFA. The data were processed statistically using Statistica ver. 13.3 software [22]. The effects of the production system and fat depots on the FA profile were evaluated by two-way analysis of variance (ANOVA) for orthogonal designs, with interactions. The significance of differences between means was estimated by Tukey’s test, at significance levels of *p* = 0.05 and *p* = 0.01.

## 3. Results

Intensively fattened bulls had higher live weight at slaughter and significantly (*p* ≤ 0.05) higher hot carcass weight and dressing percentage than semi-intensively fattened animals (Table 3). The carcasses of intensively fattened bulls scored higher for conformation (8.75 points) and somewhat lower for fatness (4.75 points) in the EUROP classification system, compared with the carcasses of semi-intensively fattened bulls (8.00 and 4.88 points, respectively). The higher fat cover of bulls in the SI production system resulted in a higher proportion of fat in the three-rib cut and higher intramuscular fat content of the carcass (1.94% vs. 1.39%).

The proportion of SFAs in fat depots was higher (*p* ≤ 0.05) in the carcasses of intensively fattened animals than in those fattened semi-intensively (Table 4.54.47 vs. 52.34). The proportions of major FA groups were also significantly (*p* ≤ 0.01) affected by fat depot. Perirenal fat and intermuscular fat contained significantly (*p* ≤ 0.01) more SFAs (58.37% and 58.10%, respectively) than intramuscular fat (49.37%) and subcutaneous fat (48.32%). Regardless of production system, intramuscular fat had a significantly (*p* ≤ 0.01) higher content of PUFAs and n-6 FAs. The concentration of UFAs and MUFAs were also significantly (*p* ≤ 0.01) higher in intramuscular and subcutaneous fat.

In most cases, fattening intensity had no significant effect on the average values of FA ratios in bovine fat, with the exception of the MUFA/SFA (*p* ≤ 0.05). However, the ratios between the analyzed FA groups differed across fat depots in both production systems. The MUFA/SFA was significantly (*p* < 0.01) higher in subcutaneous fat (0.99) and intramuscular fat (0.90) than in the remaining types of fat. The PUFA/SFA was also significantly (*p* < 0.01) higher (0.14) in intramuscular fat than in the remaining types of fat in both production systems. The content of the analyzed SFAs in bovine fat was not significantly affected by fattening intensity, but it varied across fat depots. Irrespective of production system, intramuscular fat was characterized by a significantly (*p* ≤ 0.01) lower content of C14:0, compared with the remaining types of fat. The content of stearic acid (C 18:0), regarded as the most important FA in beef, was significantly lower in subcutaneous and intramuscular fat than in perirenal and intermuscular fat.

An analysis of the effect of fattening intensity on the proportion of MUFAs in bovine fat revealed that the content of C 18:1 trans 10 + 11 and C18:1 cis11 was somewhat higher in the carcasses produced under the intensive system, compared with the semi-intensive system. The content of oleic acid (C18:1 cis9) was significantly (*p* ≤ 0.05) higher in semi-intensively fattened bulls than in animals fed diets with increased energy density. In both production systems, perirenal fat and intermuscular fat had a significantly (*p* ≤ 0.01) higher content of C18:1 trans 10 + 11, compared with subcutaneous fat and intramuscular fat, which in turn had a significantly (*p* ≤ 0.01) higher content of oleic acid and C 18:1 cis 11.

Regardless of production system, the content of PUFAs (C18:2, C18:3, C20:4, C20:5 EPA) was significantly (*p* ≤ 0.01) higher in intramuscular fat than in the remaining fat depots. At both levels of fattening intensity, subcutaneous fat was characterized by a significantly (*p* < 0.01) higher content of CLA than intramuscular fat. The concentration of PUFAs in the analyzed fat depots was not affected by fattening intensity, whereas the proportions of C 22:5 and C 22:6 were higher in the fat of intensively fattened bulls than semi-intensively fattened animals.

## 4. Discussion

In this study, intensively fattened bulls had higher live weight at slaughter and, consequently, higher hot carcass weight and dressing percentage, compared with semi-intensively fattened animals, which corroborates the findings of many authors [2,23]. Higher fattening intensity, including a higher proportion of concentrate in the ration, leads to higher average daily gain. In both production systems, bulls were slaughtered at 18 months of age, but the carcasses of semi-intensively fattened animals tended to have higher fat cover and intramuscular fat content. In the present experiment, similarly to a study by Moholisa et al. [23], higher muscle fat content in cattle corresponded to fatter carcasses as a result of feeding high-energy diets. Rodríguez-Vázquez et al. [24] observed that cattle grazing pasture had lower intramuscular fat content than those fed grain or concentrate, which is consistent with the findings of Mezgebo et al. [25] who suggested that the diet based on concentrates increases intramuscular fat content. However, the experimental animals in the cited study differed in age and final body weight from those analyzed in the current experiment. These factors may reflect the relative patterns of adipose tissue deposition, i.e., subcutaneous depot before intramuscular depot. According to Noci et al. [26], feeding intensity is one of the key factors influencing the lipid composition of meat, and a decrease in the energy density of diets may reduce muscle fat content, thus improving the quality of beef.

Differences in the proportions of FA groups, depending on feeding intensity, observed in this study, were also reported by French et al. [15]. However, the cited authors found that a decrease in the amount of concentrate and an increase in the amount of haylage in the ration for steers led to an increase in the number of SFAs in intramuscular fat. According to Turk and Smith [27], a higher percentage of SFAs in beef carcasses may result from higher C18:0 content and lower activity of Δ9-destructase. De la Fuente et al. [28] demonstrated that intensive production systems, where cattle are fed concentrate-based diets, contribute to a considerable increase in the concentrations of n-6 PUFAs in beef, and that beef produced in extensive systems has low PUFA levels and high SFA concentrations, which is partially consistent with the results of the present study. Raes et al. [29] also found that the proportions of FA groups in bovine fat may be determined by the diet. Fresh grass (pasture) and grass silage are richer sources of n-3 PUFAs and have a more favorable n-6/n-3 PUFA ratio than concentrate, which can lead to an increase in these parameters in carcass fat in cattle fed greater amounts of the former diets. Such a relationship was not observed in the current study. It appears that the fact that feeding intensity had no significant effect on the proportions of FA groups in the analyzed fat depots in bull carcasses could be due to smaller differences between the examined production systems, compared with previous studies.

According to the British Department of Health [30], the n-6/n-3 PUFA ratio should not exceed 4.0. In the present study, the values of the n-6/n-3 PUFA ratio were within this range in all types of adipose tissue and in both production systems. Bilik et al. [31] found that different feeding intensity levels in Limousin bulls had a significant effect on the n-6/n-3 PUFA ratio, which was not observed in this study.

The proportions of FA groups were significantly affected by the type of fat, which was also reported by other authors [5,7,32]. Similar to the present study, Aldai et al. [33] noted that SFA content was highest in intermuscular fat and lowest in intramuscular fat. High PUFA concentrations in intramuscular fat, compared with other types of fat, were also observed by Harper and Pethick [34], and Pethick et al. [35], who attributed this phenomenon to the small size of adipocytes. According to Nurnberg et al. [36] and Arana et al. [37], a higher ratio of phospholipids to neutral lipids contributes to a higher proportion of PUFAs in intramuscular fat. The content of MUFAs in subcutaneous fat was high in the current experiment and in previous studies [33,38,39]. Białek and Tokarz [40] suggested that it could result from increased activity of the enzyme which converts myristic acid (C14:0), palmitic acid (C16:0) and stearic acid (C18:0) to their corresponding MUFAs. According to Bartoň et al. [32], a higher proportion of SFAs in perirenal fat, compared with subcutaneous fat, resulted from a higher content of C18:0. The above authors stressed that a higher proportion of MUFAs in subcutaneous fat, observed also in the present study, could be linked with higher concentrations of C14:1, C16:1 and C18:1.

Stearic acid C18:0 belongs to the most important functional FAs. According to Oka et al. [41] and De Smet et al. [42], the percentage of stearic acid decreases in beef carcasses with an increase in their fat content. This finding was confirmed in the current study where C18:0 content was lower in the fatter carcasses of semi-intensively fattened bulls, and it was lowest in subcutaneous fat. Similar observations were made by Bartoň et al. [32]. As stearic acid is easily converted to oleic acid C18:1, it is regarded as neutral or capable of reducing blood cholesterol levels [43]. According to Gebauer et al. [44], myristic acid (C 14:0) and palmitic acid (C 16:0) exert adverse health effects by increasing the risk of cardiovascular disease and cancer. In the current study, the content of these acids was lowest in intramuscular fat. In the work of Mapiye et al. [45], the proportion of C 14:0 was higher in subcutaneous fat than in perirenal fat.

Considerable differences between FA groups point to the generally known relationships described in other studies investigating the effects of production systems and diets on the proportion of PUFAs in cattle [1,26,46,47,48]. Concentrate-based diets contribute to a higher level of n-6 C18:2 and a lower level of n-3 C18:3 and other long-chain FAs (EPA, DHA and DPA) [49,50], which was also partially confirmed in the present study.

In both this experiment and previous studies [5,7,33,51], CLA content was highest in subcutaneous fat, and its value determined in this study (0.44%) is consistent with those reported by the cited authors. According to Brugiapaglia et al. [51], CLA is preferentially incorporated into neutral lipids.

Aharoni et al. [52] demonstrated that dietary manipulation induces smaller changes in the CLA content of meat, compared with milk fat. Three mechanisms were proposed to explain this fact. First, milk fat composition reflects the composition of feed consumed by cows during the experiment, whereas only some muscle lipids are deposited over this period. Second, digesta passage rates through the rumen may be different in dairy cows characterized by very high dry matter intake, and growing animals characterized by lower dry matter intake. Third, the rate of CLA synthesis in the mammary glands may be different from that in adipose and muscle tissues [52].

## 5. Conclusions

This study demonstrated that an increase in the amount of concentrate in the ration led to an increase in the proportion of SFAs and a decrease in the proportion of UFAs in beef. Intramuscular fat was most abundant in total PUFAs, n-6 PUFAs and functional FAs such as C 18:2, C 18:3, C 20:4 and C 20:5, in comparison with the remaining types of adipose tissue. External fat was more abundant in CLA than other fat types.

## Figures and Tables

**Table 1:** Chemical composition and nutritional value of experimental diets (mean ± SD).

Specification	Silage *n* = 9	Triticale *n* = 1	Rapeseed Meal *n* = 1	Concentrate I *n* = 7	Concentrate II *n* = 7
Triticale (g/kg)				710	770
Rapeseed meal (g/kg)				250	190
Dry matter	397 ± 109.3	881	887	883.9 ± 7.1	885.5 ± 8.2
In Dry matter [g/kg]					
Organic matter	920 ± 30.6	981	927	932 ± 13.1	925 ± 18.3
Crude protein	141 ±11.4	133	388	189 ± 15.1	163 ± 7.1
NDF ^1^	569 ± 52.3	193	310	202 ± 11.2	184 ± 7.9
ADF ^2^	387 ± 59.2	44	228	72 ± 5.8	31 ± 8.2
DOMD ^3^	741 ± 55.9	932 ± 26.5	848 ± 4.4	-	-
UFV ^4^	0.80 ± 0.03	1.21	1.01	1.18 ± 0.03	1.21 ± 0.02
PDIN ^5^	82.2 ± 6.64	89	259	122.2 ± 2.4	112.4 ± 5.2
PDIE ^6^	69.5 ± 2.28	109	163	129.6 ± 5.2	121.1 ± 4.7

Fermentation characteristics of silage: pH 4.8 ± 0.3; lactic acid—54 ± 20.4; volatile fatty acids—27 ± 5.3; water-soluble carbohydrates—82 ± 47.6; N-NH_3_ g/1000 g^−1^ N—103 ± 67.4; protein nitrogen 518 ± 45.6 g/1000 g^−1^ N. ^1^ Neutral Detergent Fiber, ^2^ Acid Detergent Fiber, ^3^ Digestible Organic Matter Digestibility, ^4^ Meat Production Units,^5^ Protein digested in the small intestine depending on rumen degraded protein^, 6^ Protein digested in the small intestine depending on rumen fermented organic matter.

**Table 2:** Composition and nutritional value of the total mixed ration (TMR).

Specification	Semi-Intensive Production System <300 kg BW	Intensive Production System <300 kg BW	Semi-Intensive Production System > 300 kg BW	Intensive Production System > 300 kg BW
Grass silage (kg/100 kg)	75	60	75	60
Concentrate (kg/100 kg)	25	40	25	40
Dry matter	518.5	591.4	519.0	592.2
In Dry matter [g/kg]				
Crude protein	153	160	146	150
NDF ^1^	473	415	466	405
ADF ^2^	308	184	298	167
UFL	0.90	0.96	0.91	0.97
PDIN	92.2	98.2	89.8	94.3
PDIE	83.5	93.5	82.4	91.1
				

^1^ Neutral Detergent Fiber, ^2^ Acid Detergent Fiber.

**Table 3:** Characteristics of bull carcasses in intensive and semi-intensive production systems.

	Production System (PS)		*p*-Value
Specification	I	SI	
Live weight at slaughter, kg	544.43	507.50	0.145
Hot carcass weight, kg	312.90	280.35	0.041
Dressing percentage, %	60.49	58.15	0.032
Conformation score, EUROP system, points	8.75	8.00	0.176
Fat scores, EUROP system, points	4.75	4.88	0.876
Weight of the three-rib cut, kg			
Share in the three-rib cut, %			
fat	15.59	16.69	0.316
lean meat	58.43	56.37	0.167
bones	21.33	22.00	0.612
tendons	4.65	4.94	0.672
Intramuscular fat content, %	1.39	1.94	0.301

EUROP conformation score: 1 muscling very weak (class P−)—15 muscling outstanding (class E+). EUROP degree of fat scores: 1 none up to low fat cover (class 1−)—15 very high (class 5+).

**Table 4:** Effect of production system and type of adipose tissue on the fatty acid profile, fatty acid groups and ratios.

	Production System (PS)		Adipose Tissue (AT)				SEM	Significance		Interaction PS × AT
Specification	I	SI	SUBF	KIDF	INMF	ITMF		PS	AT	
Major fatty acid (FA) groups, % of total FAs										
SFAs	54.47	52.34	48.32 ^B^	58.37 ^A^	58.10 ^A^	49.37 ^B^	0.785	*p* ≤0.05	*p* ≤0.01	ns
UFAs	45.53	47.62	51.67 ^A^	41.57 ^B^	41.91 ^B^	50.64 ^A^	0.788	ns	*p* ≤0.01	ns
MUFAs	40.24	42.88	47.13 ^A^	37.29 ^B^	37.21 ^B^	43.94 ^A^	0.779	*p* ≤0.05	*p* ≤0.01	ns
PUFAs	5.30	4.74	4.55 ^B^	4.27 ^B^	4.70 ^B^	6.70 ^A^	0.275	ns	*p* ≤0.01	ns
n-6	3.02	2.85	2.08 ^B^	2.55 ^B^	2.62 ^B^	4.53 ^A^	0.163	ns	*p* ≤0.01	ns
n-3	1.63	1.25	1.82	1.02	1.38	1.63	0.212	ns	ns	ns
Ratios										
PUFA/SFA	0.10	0.09	0.10 ^B^	0.07 ^B^	0.08 ^B^	0.14 ^A^	0.006	ns	*p* ≤0.01	ns
MUFA/SFA	0.76	0.85	0.99 ^A^	0.66 ^B^	0.65 ^B^	0.90 ^A^	0.026	*p* ≤0.05	*p* ≤0.01	ns
n-6/n-3	2.53	2.32	1.93 ^B^	2.61 ^A^	2.47 ^A^	2.75 ^A^	0.084	ns	*p* ≤0.01	ns
Saturated fatty acids (SFAs), g/100 g										
C 14:0	3.09	2.94	3.25 ^A^	3.13 ^A^	3.21 ^A^	2.49 ^B^	0.076	ns	*p* ≤0.01	ns
C 16:0	26.77	26.39	27.66	26.38	26.56	25.83	0.255	ns	ns	ns
C 18:0	21.43	20.01	14.73 ^B^	25.27 ^A^	24.75 ^A^	18.49 ^B^	0.766	ns	*p* ≤0.01	ns
Unsaturated fatty acids (UFAs), g/100 g										
C 18:1 T 10 + 11	2.23	2.09	1.86 ^B^	2.68 ^A^	2.65 ^A^	1.48 ^B^	0.098	ns	*p* ≤0.01	ns
C 18:1 C9	30.71	33.24	34.82 ^A^	28.02 ^B^	28.60 ^B^	35.82 ^A^	0.619	*p* ≤0.05	*p* ≤0.01	ns
C 18:1 C11	1.37	1.33	1.47 ^A^	1.34 ^AB^	1.19 ^B^	1.41 ^AB^	0.031	ns	*p* ≤0.01	ns
C 18:2	2.74	2.56	1.99 ^B^	2.45 ^B^	2.54 ^B^	3.67 ^A^	0.118	ns	*p* ≤0.01	ns
C 18:3	0.75	0.74	0.61 ^B^	0.72 ^B^	0.72 ^B^	0.92 ^A^	0.026	ns	*p* ≤0.01	ns
CLA	0.37	0.40	0.44 ^A^	0.40 ^A^	0.40 ^A^	0.28 ^B^	0.011	ns	*p* ≤0.01	ns
C 20:4	0.25	0.26	0.06 ^B^	0.07 ^B^	0.05 ^B^	0.82 ^A^	0.050	ns	*p* ≤0.01	ns
C 20:5 EPA	0.07	0.10	0.06 ^B^	0.06 ^B^	0.06 ^B^	0.15 ^A^	0.011	ns	*p* ≤0.01	ns
C 22:5 DPA	0.47	0.14	0.62	0.06	0.28	0.35	0.147	ns	ns	ns
C 22:6 DHA	0.23	0.07	0.26	0.06	0.21	0.08	0.059	ns	ns	ns

I—intensive production system; SI -semi-intensive production system; SUBF—subcutaneous fat, KIDF—kidney fat, INMF—intermuscular fat, ITMF—intramuscular fat; CLA—C 18:2 cis 9 trans 11. mean values within the production system and adipose tissue denoted by different letters are significantly different at: A,B—*p* ≤ 0.01; ns—not significant.

## Data Availability

The data presented in this study are available on request from the corresponding author.
